# An eight-week placebo-controlled RCT on the efficacy of a probiotic nutritional intervention for subclinical gastrointestinal symptoms in students

**DOI:** 10.1038/s41598-026-44433-4

**Published:** 2026-03-19

**Authors:** Alexander Winkler, Christiane Hermann, Alannah Hahn, Daniel Beis

**Affiliations:** 1 Department of Clinical Psychology and Psychotherapy, Justus-Liebig-University, Otto-Behaghel-Str. 10F, 35394 Giessen, Germany; 2 Department of Agricultural Sciences, Nutritional Sciences, and Environmental Management, Institute of Nutrition Sciences, Justus-Liebig-University, Giessen, Germany

**Keywords:** Probiotics, Gastrointestinal, Placebo, Stress, Treatment, Psychology, Gastroenterology

## Abstract

Despite evidence for substantial placebo effects in the treatment of gastrointestinal (GI) complaints, there is still no methodologically sound study on placebo effects in their probiotic treatment. This randomized controlled trial (RCT) aimed to determine the contribution of a placebo effect in probiotic fruit bit intake in a sample of individuals with mild to moderate gastrointestinal complaints. A sample of 83 individuals were randomly assigned to a probiotic, placebo or to a no-intervention group. All participants received the same information, and the intervention groups took the respective fruit bits daily for eight weeks. Before and after the intervention gastrointestinal symptoms and burden were assessed. Participants in the probiotic (*d* = -1.08) and placebo group (*d* = -1.17) benefitted significantly more compared to the no-intervention group with respect of gastrointestinal symptoms, but no significant difference was seen between the probiotic and the placebo group (*d* = 0.04). At the end of the intervention phase, no significant group differences regarding stress, somatization, and well-being were noted. In both intervention groups, self-reported treatment expectation was not correlated with symptom improvement. Interestingly, in the probiotic group only, subjectively perceived improvement correlated significantly with the change in GI symptoms. While individuals benefitted from a novel probiotic food supplement, data indicated a substantial placebo response. Explicit treatment expectation seems to play a minor role in accounting for the observed intervention effects, yet might contribute to continued use of probiotics.

## Introduction

Millions of people worldwide suffer from gastrointestinal complaints^1^. In addition to gastrointestinal diseases (e.g., inflammatory bowel disease), many individuals experience gastrointestinal symptoms without any underlying known physiological anomalies to explain their symptoms^2, 3^. Such symptoms are now classified as disorders of gut-brain interaction (DGBI), formerly known as functional gastrointestinal disorders (FGID), with irritable bowel syndrome (IBS) and functional dyspepsia (FD) as the most frequent ones, affecting more than 40% of the population according to a recent study by the Rome Foundation^4^. Individuals suffering from DGBI may feel stigmatized, and diagnoses are often poorly communicated by physicians or neglected due to the absence of detectable structural changes^2^. Consequently, the actual number of individuals with mild to moderate gastrointestinal complaints without having been appropriately diagnosed is likely to be higher.

Despite the unclear clinical evidence, probiotic dietary supplements have become hugely popular for improving gastrointestinal symptoms and gastrointestinal well-being. They are widely available online, in drugstores, and even in supermarkets, leading to a pronounced increase in their use, with global retail sales projected to reach $11.53 billion by 2028^5^. Probiotics are defined as living microorganisms that, when administered in adequate amounts, confer a health benefit on the host, a definition widely recognized since 2001 by the FAO and WHO^6^. These microorganisms, predominantly bacteria from the genera Lactobacillus and Bifidobacterium, as well as other genera (e.g. Bacillus, Escherichia) and other microorganisms (e.g. yeast Saccharomyces boulardii), typically originate from fermented foods or the human gut flora and have been used in various products for decades^7^.

According to the Global Guidelines: Probiotics and Prebiotics^7^, recent findings suggest that probiotic interventions may have a positive effect on individuals with DGBI. Studies combining probiotic strains of Lactobacillus and Bifidobacterium have reported significant improvements in stress-related and functional gastrointestinal symptoms, although not always being superior to the placebo group^8, 9^. Probiotic intake may modulate gut microbiota composition and influence perceived stress levels, linking the reduction of stress to improved gastrointestinal health^10, 11, 12^. Interestingly, in a large sample of non-patients with subclinical GI symptoms, an intervention with probiotic bifidobacterium infantis was more effective than placebo^13^. However, the heterogeneity of study designs and the strain- and dose-specific properties of the bacteria limit the comparability of existing studies^7^. Hence, research is needed to better understand the specific mechanisms of probiotic efficacy^14, 15, 16^. The lack of consistent differences in symptom improvement between placebo and probiotic interventions might suggest a substantial placebo component of probiotics. Thus far, this has not been investigated experimentally while taking into account the natural course of GI symptomatology.

Enck and colleagues reviewed the placebo arms of 16 meta-analyses, which included over 600 randomized, placebo-controlled trials for gastrointestinal disorders^17^. The placebo response rate (i.e. number of people responding to placebo condition) in IBS patients was on average between 30% and 40%, but lower in gastrointestinal diseases (15% to 30%). It was also noted that, across studies, the findings are flawed due to methodological inconsistencies^17^. Specifically, studies differ considerably with regard to treatment duration, sample size, symptom severity at baseline, frequency of clinician-patient interaction, and inclusion criteria. Moreover, placebo response rates depend on the success criterion (i.e., full remission vs. symptom improvement). Furthermore, improvements resulting from placebo treatment may also be due to spontaneous remissions^17^. In order to distinguish between a placebo effect and the natural course of the disorder, an untreated control group (“no-intervention group”) is needed. However, such a control group is unethical in serious GI diseases (e.g., ulcerative colitis, Crohn’s disease). Intervention studies in IBS have rarely included such a control condition^17^. Kaptchuk et al.^18^ randomly assigned a total of 80 severely affected patients with IBS (Rome III criteria) to either an open-label placebo for three weeks or a no treatment condition. The placebo was presented as an “inert substance similar to a sugar pill that can contribute to significant symptom improvement through the self-healing process of mind and body,” to be taken twice daily. Patients in the open-label placebo group experienced a significantly greater overall symptom improvement and enhanced quality of life compared to the control group^18^. In a subsequent study^19^, a total of 262 IBS patients either underwent an open-label or double-blind placebo or no intervention. In both placebo group, IBS symptoms improved significantly compared to the no- intervention group, with no significant differences between the placebo groups. Patients’ expectations and conditioning experiences presumably account for the placebo effect in gastrointestinal treatments ^20, 21^. Most of what is known about the placebo effect in the treatment of gastrointestinal disorders comes from meta-analyses primarily of placebo-controlled drug trials^17, 22^.

The aim of the current study was to experimentally determine the proportion of the placebo effect in accounting for the efficacy of a probiotic treatment of subclinical gastrointestinal complaints (i.e., without a clinical diagnosis). Specifically, a randomized parallel group design, comparing the probiotic intervention to a no-intervention and a placebo group was used. Participants in the probiotic and placebo group underwent an eight-week intake of a novel probiotic food containing either the combination of two strains of the aforementioned probiotic bacterial genera or not containing probiotics, whereas the no-intervention group did not receive an intervention (except participating in the weekly surveys as the other two groups). Our a priori hypotheses were that in the probiotic group, changes in the primary outcomes (GI symptoms) would be significantly greater than in the placebo and the no-intervention group. Moreover, we assessed whether placebo effects and treatment expectations may account for the putative benefits of probiotics in a largely neglected group of individuals suffering from mild to moderate gastrointestinal complaints.

## Method

### Participants

**Fig. 1:**
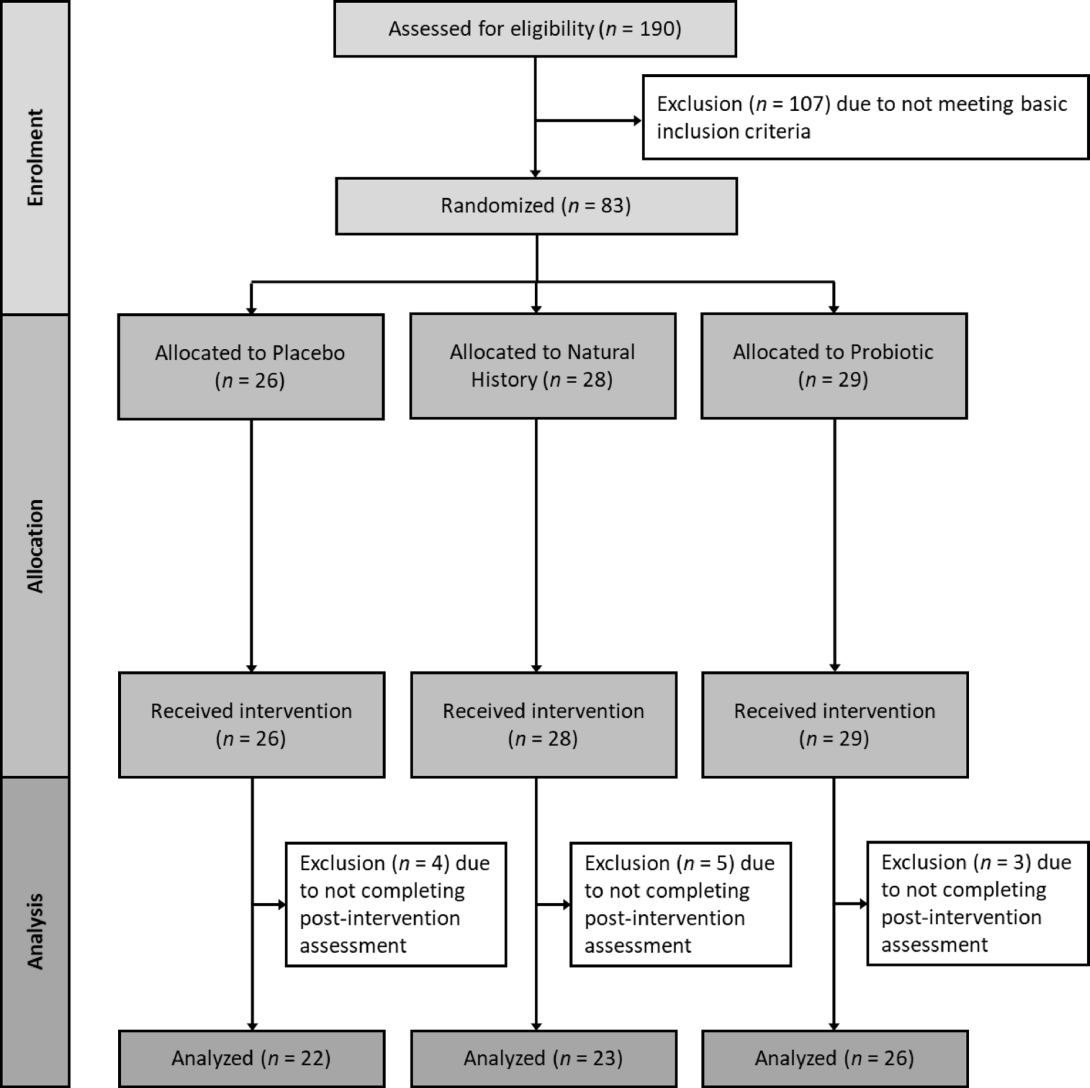
Participant flow chart.

One hundred and ninety students (76.2% females) of a German university aged between 18 and 40 years (*M* = 25.30, *SD* = 4.90) were interviewed after they had responded to an e-mail advertising a study on a probiotic treatment for gastrointestinal symptoms. Additionally, posters and flyers were displayed and distributed in various campus areas, as well as in city areas. Moreover, the study was advertised in local social networks. The following inclusion criteria were assessed via a phone interview: (a) age between 18 and 40 years; (b) mild to moderate gastrointestinal complaints, which the individual perceived as distressing and interfering with daily life and eating behavior, but was not severe enough to meet the diagnostic criteria of gastrointestinal disorder by a medical expert; and (c) fluency in German. Exclusion criteria were (a) allergies to any fruit, (b) known/diagnosed chronic or acute physical or mental illness, (c) ongoing pharmacological or psychotherapeutic treatment, (d) regular or recent (i.e.,  within the last four weeks) intake of antibiotic or probiotic medication, (e) a body mass index below 17.5 or above 35 and (f) excessive consumption of alcohol or drugs. A sample size of *N* = 164 was calculated based on an a priori power analysis (G*Power 3.1.9.2) for repeated measures, and the interaction effect between groups (probiotic, placebo, no-intervention group) and time (pre- and post-intervention) to target group differences in change of gastrointestinal symptoms, assuming a small effect size of *f* = 0.15 with a power of 90% (α = 0.05, *r*=.50). Assuming a dropout rate of about 15%, *N* = 190 were interviewed at the start of the study (for an overview, see Fig. 1). All participants were informed about the study procedure and gave written informed consent. Study participants received a doybag of probiotic fruit bits at the end of the study and had the chance to win a 50€ voucher for a local bookstore. The experiment was conducted according to the Declaration of Helsinki and approved by the local ethics committee (Die lokale Ethik-Kommission des Fachbereichs 06 der Justus Liebig Universität Giessen; 2022-0017). The study was pre-registered at the German Clinical Trials Register (DRKS) on 02/08/2022 (DRKS-ID: DRKS00029742; https://drks.de/search/de/trial/DRKS00029742). Participants were consecutively, but randomly assigned in a triplet fashion to one of the groups. Specifically, triplets of participants were formed such that they were matched for biological sex (male or female) and predominant gastrointestinal symptoms (diarrhea, constipation, or bloating/abdominal pain). For example, three female participants primarily suffering from diarrhea were randomly assigned to receive the doybag for one of the two interventions or no doybag. The data collection took place from 09/2022 to 09/2023.

### Outcome Measures

#### Gastrointestinal symptoms and gastrointestinal well-being (primary outcomes)

Gastrointestinal symptoms were assessed using the German translation (developed by AstraZeneca, 1995) of the Gastrointestinal Symptom Rating Scale (GSRS), a disease-specific instrument designed to assess commonly reported gastrointestinal symptoms^23^. The scale consists of 15 items that are grouped into five symptom clusters: reflux (items 2 and 3), abdominal pain (items 1, 4, and 5), indigestion (items 6–9), diarrhea (items 11, 12, and 14), and constipation (items 10, 13, and 15). The items were selected based on clinical experience and literature reports of gastrointestinal symptoms in patients with IBS or peptic ulcer disease^23^. Respondents rate the experienced severity of their symptoms in the past week on a 7-point Likert scale, ranging from “not at all” to “very severe”, whereas higher scores indicate more severe symptoms^24^. The present version is a self-administered questionnaire that was modified by Dimenäs et al.^25^ from the original interview-based version. The four symptom clusters of diarrhea, constipation, indigestion, and reflux of the GSRS have high internal consistency with Cronbach’s alpha values between 0.70 and 0.91, while the symptom cluster of abdominal pain only showed moderate internal consistency (Cronbach’s alpha = 0.53;^26^. In the current sample, Cronbach’s alpha was 0.80 for this symptom cluster. The test-retest reliability of the GSRS ranges from 0.49 (abdominal pain) to 0.73 (indigestion;^26^, with only the two symptom clusters of indigestion and constipation reaching acceptable values (intraclass correlation coefficient > 0.70;^27^. Regarding the construct validity of the GSRS, it was shown that the relevant domains of the GSRS correlated significantly with those of the Quality of Life in Reflux and Dyspepsia (QOLRAD) questionnaire^26^. Additionally, the GSRS differentiates between patients with different severities of symptoms^28^. Overall, reliability and validity of the German version of the GSRS are satisfactory for the assessment and differentiation of gastrointestinal symptoms^26^.

*Gastrointestinal well-being* was assessed weekly using a self-developed gastrointestinal tract (GIT) questionnaire to assess not only intensity (as the GSRS), but also frequency of gastrointestinal symptoms and the associated quality of life. The GIT questionnaire is based on previously validated questionnaires on quality of life and gastrointestinal burden, including the GSRS by Svedlund et al.^23^ and the Gastrointestinal Quality of Life Index (GIQLI) by Eypasch et al.^29^, which focus on patient samples, respectively. For this study, we relied on the 10 items assessing the frequency and experienced intensity of various gastrointestinal symptoms, such as bloating and constipation, within the last week. Participants assess symptom frequency using a 5-point Likert scale (ranging from “never” to “very often/daily”), while symptom intensity was rated on a visual analogue scale (VAS). Similar to other composite measures such as the headache index, frequency and intensity were multiplied for each GI symptom and averaged, thus yielding a mean gastrointestinal index of all symptoms (i.e., GIT index). The psychometric properties of the GIT questionnaire have not yet been formally evaluated. In the current sample, Cronbach’s alpha was 0.72 for the GIT index.

#### Stress, somatization and well-being (secondary outcomes)

Psychological stress was assessed using the *Perceived Stress Scale-10* (PSS-10;^30, 31^, a widely used instrument for assessing experienced stress within the last month. For this study, we adjusted the interval to the “past two weeks” allowing a more detailed observation of change during our intervention. The PSS-10 comprises 10 items, measuring how unpredictable, uncontrollable and/or overloaded participants felt rated on a 5-point Likert scale from “never” (0) to “very often” (4), resulting in a score ranging from 0 to 40. Cronbach’s alpha of the original PSS-10 was published as being α = 0.78^31^.

Physiological stress symptoms were assessed using the respective subscale of the *Depression Anxiety and Stress Scale 21* (DASS-21^32^, shortened and translated into German^33^. The DASS-21 is a 21-item self-report measure of state negative affects in the past two weeks, developed with the specific aim of differentiating between depressive symptoms, anxiety and tension/stress. Each subscale comprises seven statements to be answered on a 4-point Likert scale ranging from “Did not apply to me at all” (0) to “Applied to me very much, or most of the time” (3), resulting in a sum score of 0–21 for each subscale. The German DASS-21 scale has good convergent and discriminant validity and high internal consistency (Cronbach’s α = 0.91 – depressive symptoms subscale, α = 0.82 – anxiety subscale, α = 0.89 – stress subscale;^33^).

Somatization symptoms were assessed using the German translation^34^ of the *Patient Health Questionnaire 15* (PHQ-15^35^. The PHQ-15 is a 15-item self-report measure assessing the severity of various somatic symptoms (e.g., pain, shortness of breath, feeling faint). The time interval was also adjusted to two-weeks, thus participants rated how much they had been affected by each symptom in the past two weeks from “not bothered at all” (0) to “bothered a lot” (2). Sum scores range from 0 to 30. The German translation of the PHQ-15 has a good internal reliability of Cronbach’s α = 0.79^36^.

Mental well-being was assessed using the German version^37^ of the *Warwick–Edinburgh Mental Wellbeing Scale (WEMWBS;*^38^). This consists of 14 positively worded statements covering subjective well-being and psychological functioning within the last two weeks. The scale is scored by summing up the response to each item answered on a 5-point Likert scale from “none of the time” (1) to “all of the time” (5), thus ranging from 14 to 70. The German version of the WEMWBS has demonstrated high reliability and validity with an internal consistency of α = 0.92^39^.

#### Treatment expectations and perceived improvement (exploratory outcome)

Treatment expectations and perceived improvement were assessed with the *Generic rating scale for previous treatment experiences*,* treatment expectations*,* and treatment effects (GEEE;*^40^. In addition to patients’ treatment-related expectations (“How much improvement do you expect from the treatment”), the GEEE assesses current experiences of treatment-related effects (“How much improvement have you experienced since your participation in this study?”). Subjects rated treatment expectations and current experiences using Numeric Rating Scales (NRS) from 0 to 10. The GEEE is currently under further evaluation in a series of different studies.

### Probiotic- and placebo-intervention

In this study, a probiotic fruit bit (40% mango, glucose syrup, sugar, thickener sodium alginate), containing two bacterial strains (Bifidobacterium animalis subsp. lactis BS01 and Lactobacillus acidophilus LA02, 2.3 × 10^9^ active fluorescent units (AFU) uptake per day) was used, which was provided by an external manufacturer of natural ingredients for the food, beverage, and nutrition industries.

An inactive placebo in the form of the same fruit bit was technically produced analogously to the probiotic bit, without the probiotic bacterial additives. Both products (the probiotic fruit bit and the placebo fruit bit) met food law standards, confirmed by the manufacturer’s quality assurance measures. The fruit bit was packed in brown packages (doybags) containing 75 g each. The package had a label with the best-before date, dosage recommendation, subject code, Med-ID for later assignment of group membership, and the contact addresses of the study leaders. Participants were instructed to take a daily portion of 5 g using a measuring spoon at the same time each day. The portion size had an energy content of about 17–18 kilocalories, therefore not substantially increasing the overall kilocalories consumption. Participants received two packages at baseline and another two packages after four weeks of participation, each lasting for two weeks. For the eight-week study participation, they received a total of four packages.

Before handing out the fruit bits, participants in the test and placebo groups were informed that the packages could either contain probiotics or not, with a 50% probability of receiving a placebo. To educate about the fruit bit, referred to as Fruit Crunchies in the study, the test and placebo groups were shown a brief information sheet about the expected effects of probiotics on gut health, the composition of the fruit bits, and some consumption possibilities. The no-intervention group got the prospect of receiving the same number of probiotic packages and the same information at the end of study.

#### Experimental setup

**Fig. 2:**
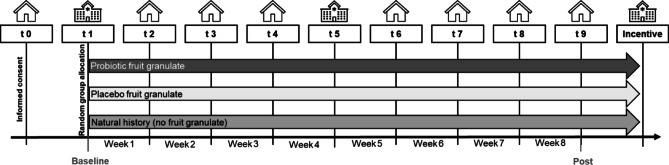
Experimental design.

The study design was a triple-blind randomized controlled trial with between-group factor group (probiotic, placebo, and no-intervention group), differing only in the consumption of the food product over the eight-week intervention period. During this period, ten measurement points (t0 – t9, within group factor) were conducted. For a schematic overview of the study design, see Fig. 2.

For the block-like randomized allocation at the first on-site appointment, six boxes of doybags were placed, i.e., three for the main GI symptom groups for each sex. The experimenters queried the subject’s sex and main symptom. Then the first pair of doybags of the respective box were handed to the subject from a randomly compiled triplet (pair of probiotics or pair of placebo doybags, or a card indicating the assignment to the no-intervention group) of packages in the box. Thus, subjects and experimenters were double-blinded regarding the group assignment to the probiotic or placebo group. The assignment was also hidden from the researcher analyzing the data. To investigate the placebo and treatment effect, this study focuses on comparing outcomes at t1 (pre treatment) and t9 (post treatment).

### Study procedure

The investigators responded to interested participants’ e-mails with a telephone interview to check the inclusion and exclusion criteria and, if suitable for the study, arranged an appointment on site. Subsequently, the online preliminary survey took place (t0). For this, participants received an invitation by e-mail for a 20 min questionnaire package via SoSci Survey by e-mail and had to give their consent to participate in the survey at the beginning. An information sheet and a written consent form for the online questionnaire survey were provided.

Approximately one to two weeks later, the first on-site examination took place at the university (t1). The on-site appointment lasted about 60 min and was performed in the examination laboratory at the Interdisciplinary Research Center (IFZ), University of Giessen. Due to ongoing Covid-19 regulations, medical masks were worn throughout the examination. At the beginning, participants were introduced to the study procedures, and the experimenters created a positive and pleasant conversational atmosphere allowing participants to ask questions and express concerns at any time. Additionally, participants were asked informal questions, such as “Do you feel healthy today or do you have any complaints?“, with the experimenters actively listening and providing empathetic understanding through regular eye contact, nodding, and friendly smiling.

Participants then received an information sheet about the study and the consent form for participation. The examination only began after written consent was obtained with a generation of a subject code for data anonymization, as well as the random group assignment. The first half of the questionnaire package then started on the computer via SoSci Survey, followed by the measurement of resting blood pressure and heart rate on the dominant arm (by an OMRON M3 upper arm blood pressure monitor). Afterward, body height, weight, and hip and waist circumference were measured. The second half of the digital questionnaire package started subsequentially. For the no-intervention group, the appointment ended after completing the questionnaires. The probiotic and placebo groups however, received their first two packages of the fruit bits, sufficient for regular consumption over four weeks. The experimenters finally gave a brief introduction to the intake and dosing of the daily portion as well as storage conditions. Experimenters explicitly asked all participants to return the two packages when coming back to the next on-site appointment for weighing. They were also instructed to regularly fill in the weekly questionnaires online. The on-site schedule was tracked using a pre-arranged case report format filled by the experimenter for each participant.

In the following three weeks, participants answered 10 min. online surveys weekly (t2, t3, and t4), with reminders sent via e-mail using the serial mail function of SoSci Survey. During this time, participants in the probiotic and placebo groups were instructed to consume the prescribed portion of the food product daily.

The second on-site appointment (t5; 30 min) took place exactly four weeks after the first appointment in the same room as before, starting with welcoming the participants in a positive and pleasant conversational atmosphere. Participants were asked how they were feeling and how their gastrointestinal complaints had changed over the past four weeks. Another online questionnaire package was then started on the computer via SoSci Survey. For the no-intervention group, the appointment ended after completing the questionnaires, while the probiotic and placebo groups returned their doybags with the remaining amount of fruit bits for measurement and they received the next two doybags for the upcoming four weeks. Participants received another briefing on consumption, dosing, and storage of the food product. A final appointment was scheduled for collecting the compensation at the end of the study, during which participants were asked to bring the new packages for weighing.

In the following four weeks, weekly online surveys via SoSci Survey continued (t6, t7, t8, and t9). After the final assessment (t9) participants had to attend a final on-site appointment at the university to return the remaining amount of fruit bits and collect their compensation. While participants in the probiotic and placebo groups received an additional package of the probiotic food product, the no-intervention group received four packages as compensation for the previous weeks together with the product information sheet and product questionnaire as well as an additional doybag as benefit.

A case report form was filled out for the on-site appointments to document the procedure of each appointment to ensure standardization of the appointments. A protocol was also used for the compensation collection appointment to ensure standardization until the end of the study. Both experimenters practiced the procedure in advance during pilot trials and received feedback from the other experimenter, thus maximizing standardization.

### Statistical analysis

Statistical analyses were performed with IBM SPSS Statistics 29.0 for Windows (IBM, Armonk, NY/USA). The significance level was set at α = 0.05 and tests were two-tailed. Differences in baseline sample characteristics were analyzed using univariate analysis of variance (ANOVA) and χ²-tests. To assess group differences in primary and secondary outcomes, a 3 × 2 mixed ANOVA with group as between-factor (probiotic, placebo, no-intervention group) and time as within-factor (pre- vs. post- treatment) was conducted. A significant group x time interaction in the ANOVA was followed up by planned contrasts comparing probiotic vs. placebo fruit bits (contrast 1), placebo fruit bits vs. no-intervention group (contrast 2) and probiotic fruit bits vs. no-intervention group (contrast 3). Cohen’s *d* was used as measure of effect size, following Cohens^41^ interpretation for *d* = 0.20 (small effect), *d* = 0.50 (medium effect), and *d* = 0.80 (large effect). Since less than 15% of participants did not complete the post-treatment assessment, an intent-to-treat analysis was not considered necessary. The proportion of symptom reduction in the GIT attributable to placebo was calculated by dividing the effect size of symptom reduction in the placebo group by the effect size of symptom reduction in the probiotic group. Additionally, for each participant, the Reliable Change Index (RCI)^42^ was calculated for all primary outcomes and compared between groups using χ²-tests, with a RCI > 1.96 threshold for classification of responders. The RCI is considered a measure of clinical significance as it indicates whether an observed change in symptoms is greater than the measurement error. Calculating the RCI requires information about the reliability of a measure. We used the reliability coefficient as determined at baseline (GSRS: Cronbach’s α = 0.80; GIT: Cronbach’s α = 0.72). As suggested by Jacobson and Truax^42^, individuals with a RCI > 1.96 can be classified as responders.

## Results

For the following analysis, one hundred and seven participants were excluded from the one hundred and fifty participants, due to the exclusion criteria. Twelve participants were excluded due to not completing the post-intervention assessment. Following the block-like randomization, the groups did also not differ concerning age, number of females, body weight, stress and gastrointestinal symptoms (see Table 1).

**Table 1:** Sample characteristics and group differences at baseline.

	Placebo (*n* = 26)	No-intervention group (*n* = 28)	Probiotic (*n* = 29)	Group differences
Age in years, M (SD)	26.23 (5.65)	25.32 (4.95)	24.48 (4.14)	*F*(2,80) = 0.87, *p* = .425
Number of females, n (%)	20 (76.9%)	22 (78.6%)	22 (73.3%)	χ²(2) = 0.06, *p* = .970
Body weight, *M* (*SD*)	70.56 (12.97)	71.30 (13.25)	66.11 (11.84)	*F*(2,81) = 1.42, *p* = .247
PSS, *M* (*SD*)	16.96 (3.49)	18.21 (6.15)	18.67 (5.54)	*F*(2,81) = 0.78, *p* = .462
GSRS, *M* (*SD*)	3.25 (0.91)	3.03 (0.79)	3.01 (0.77)	*F*(2,81) = 0.69, *p* = .505
GIT, *M* (*SD*)	218.36 (80.78)	218.21 (75.62)	221.90 (90.50)	*F*(2,81) = 0.18, *p* = .982
GEEE, *M* (*SD*)	7.19 (2.33)	-	6.77 (2.60)	*F(1*,*54) = 0.41*,* p = .524*

*Note. M* = mean; *SD* = standard deviation; *n* = number of participants; PSS = Perceived stress scale (t1); GSRS = Gastrointestinal Symptom Rating Scale (t1); GIT = Gastrointestinal Questionnaire (t1); GEEE = treatment expectation (t1).

### Effect on gastrointestinal symptoms and gastrointestinal index as primary outcomes

With regard to gastrointestinal symptoms monitored with the GSRS questionnaire, the 3 × 2 ANOVA yielded a significant interaction effect group x time (*F*[2, 68] = 9.55, *p* < .001, see Table 2). Overall, gastrointestinal symptoms decreased from pre- to post-intervention (main effect time: *F*[1, 68] = 106.05, *p* < .001). Groups did not differ significantly (main effect group: *F*[2, 68] = 1.58, *p* = .213). In terms of gastrointestinal index assessed with the GIT questionnaire, the 3 × 2 ANOVA yielded a significant interaction effect group x time (*F*[2, 68] = 4.43, *p* = .016, see Table 2). Moreover, there was a significant reduction in gastrointestinal index (main effect time: *F*[1, 68] = 22.68, *p* < .001). There was no significant main effect of group (*F*[2, 68] = 0.68, *p* = .510).

**Table 2:** Primary and secondary outcomes at baseline and post-intervention per group, main effects (group, time), and group x time interaction.

	Placebo (*n* = 22)				No-intervention group (*n* = 23)				Probiotic (*n* = 26)				Main effect group		Main effect time		Group x time interaction	
	Baseline assessment		Post assessment		Baseline assessment		Post assessment		Baseline assessment		Post assessment							
	M	SD	M	SD	M	SD	M	SD	M	SD	M	SD	F (2,68)	*p*	F (1,68)	*p*	F (2,68)	*p*
Primary outcomes																		
Gastro. symptoms (GSRS)	3.12	0.86	2.12	0.63	3.02	0.74	2.72	0.10	2.99	0.74	2.03	0.63	1.58	0.213	106.05	< 0.001	9.55	< 0.001
Gastro. index (GIT)	206.45	80.38	161.76	70.76	214.15	75.95	207.85	88.16	223.84	92.47	161.66	101.09	0.68	0.510	22.68	< 0.001	4.43	0.016
Secondary outcomes																		
Psych. stress (PSS-10)	16.36	3.43	17.05	6.13	17.83	5.85	15.30	7.10	18.42	5.85	18.15	7.25	0.84	0.436	0.85	0.360	1.50	0.231
Somatization (PHQ-15)	26.76	5.39	23.38	5.40	27.00	4.22	25.43	3.37	24.81	4.37	22.81	4.02	2.01	0.142	39.57	< 0.001	2.08	0.132
Physio. stress (DASS-21)	14.14	3.94	12.73	4.29	13.61	4.54	13.91	4.27	15.00	4.20	13.46	4.52	0.28	0.759	3.07	0.084	1.39	0.257
Well-being (WEMWBS)	48.36	6.48	49.45	8.30	50.00	6.67	48.22	9.08	48.46	8.03	45.92	9.26	0.50	0.608	1.98	0.164	2.05	0.136

Note. *M* = mean; *SD* = standard deviation; *n* = number of participants; GSRS = Gastrointestinal Symptom Rating Scale; GIT = Gastrointestinal Questionnaire; PSS-10 = Perceived Stress Scale (ranging from 0 to 40); DASS-21 = Depression Anxiety and Stress Scale (each scale ranging from 0 to 21); PHQ-15 Somatization scale from the Patient Health Questionnaire (ranging from 0 to 30); WEMWBS = Warwick–Edinburgh Mental Wellbeing Scale (ranging from 14 to 70).

**Fig. 3:**
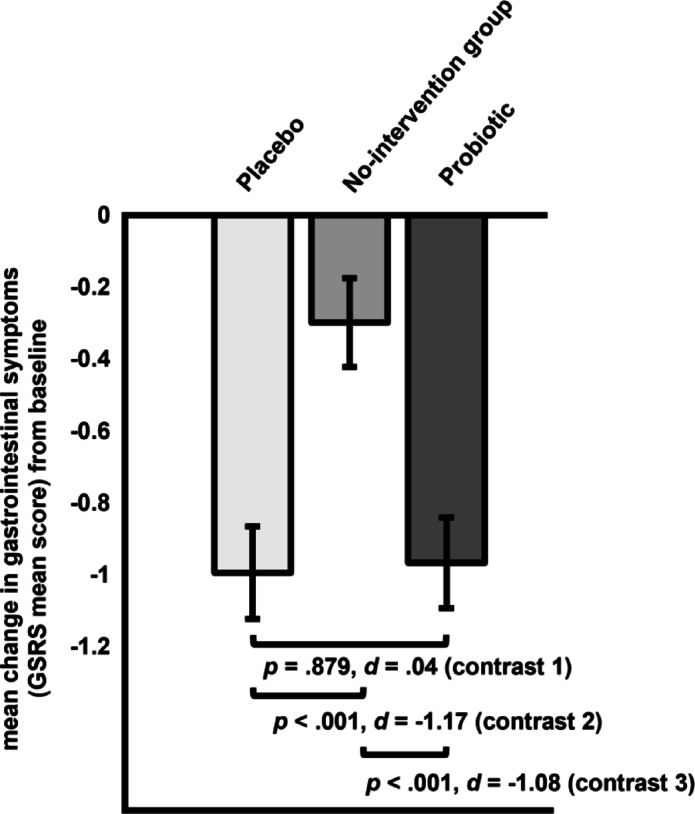
Planned contrasts for change in gastrointestinal symptoms (*GSRS*) from pre- to post-intervention for all groups (contrast 1: placebo vs. probiotic; contrast 2: placebo vs. no-intervention group; contrast 3: probiotic vs. no-intervention group).

*Gastrointestinal symptoms (GSRS).* Planned contrasts comparing probiotic vs. placebo fruit bits (contrast 1) yielded no difference in gastrointestinal symptoms change scores (∆ _probio – placebo_= 0.27, *SE* = 0.18, *p* = .879, *d* = 0.04; see Fig. 3). Gastrointestinal symptoms decreased significantly more in the placebo fruit bits vs. the no-intervention group (contrast 2: ∆ _placebo – no−intervention_= -0.70, *SE* = 0.18, *p* < .001, *d* = -1.17). Similarly, the probiotic group showed significantly greater improvement than the no-intervention group (contrast 3: ∆ _probio – no−intervention_= -0.67, *SE* = 0.18, *p* < .001, *d* = -1.08).

**Fig. 4:**
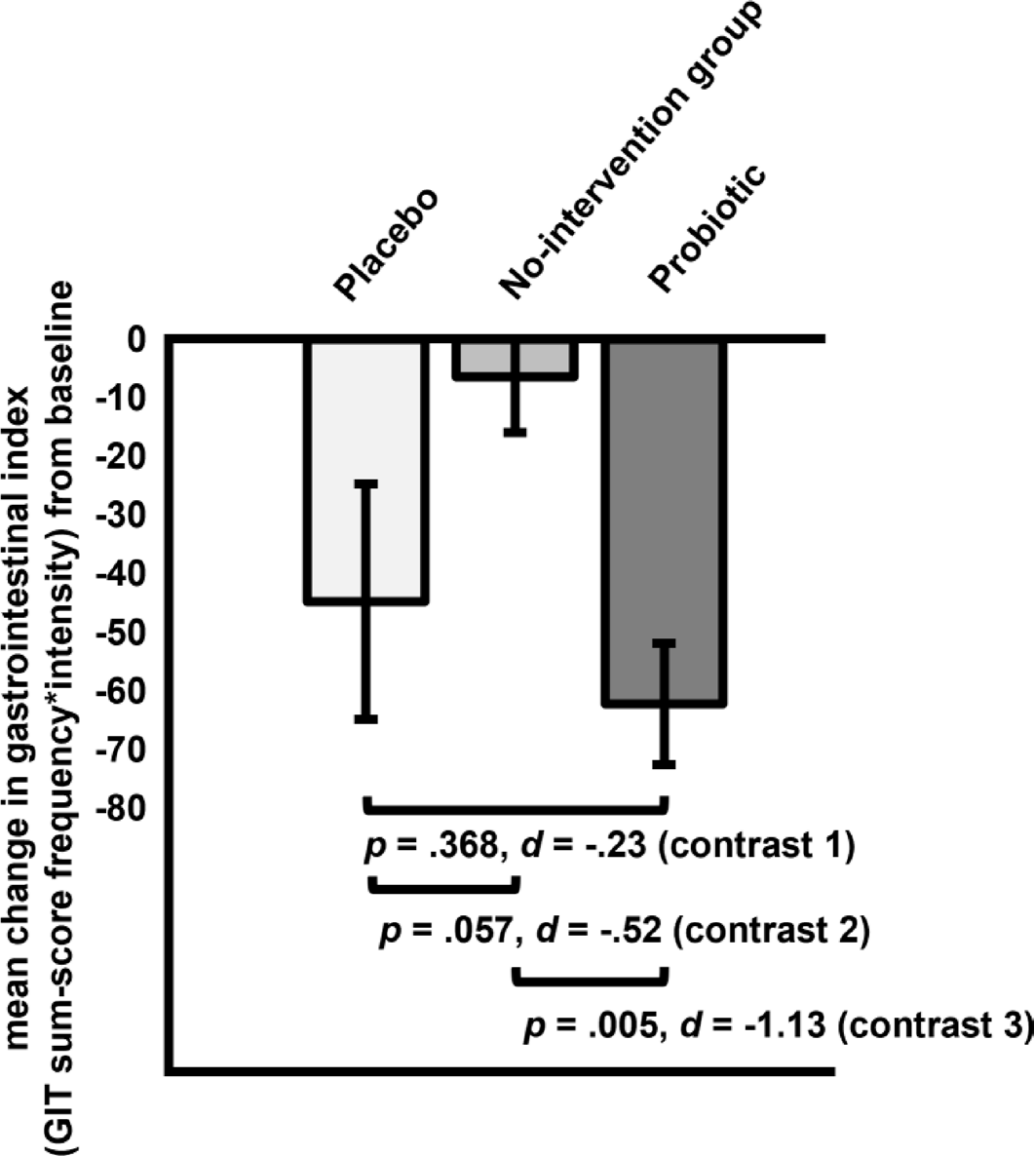
Planned contrasts for change in gastrointestinal index (*GIT*) from pre- to post-intervention for all groups (contrast 1: placebo vs. probiotic; contrast 2: placebo vs. no-intervention group; contrast 3: probiotic vs. no-intervention group).

*Gastrointestinal index (GIT)*. Planned contrasts comparing probiotic and placebo fruit bits (contrast 1) yielded no difference in gastrointestinal index change scores (∆ _probio – placebo_= -17.50, *SE* = 19.28, *p* = .368, *d* = − 0.23; see Fig. 4). There was no significant difference in the symptom reduction of the gastrointestinal index between the placebo and the no-intervention group, even though the effect size is moderate in magnitude (contrast 2: ∆ _placebo – no−intervention_= -38.39, *SE* = 19.85, *p* = .057, *d* = − 0.52). Also, participants taking the probiotic fruit bits showed a significantly greater reduction in the gastrointestinal index compared to the no-intervention group (contrast 3: ∆ _probio – no−intervention_ = -55.88, *SE* = 19.06, *p* = .005, *d* = -1.13). The proportion of symptom improvement in the probiotic group explained by the symptom reduction observed in the placebo group was 46.02%.

*Reliable change.* The proportion of participants within each group with a Reliable Change Index (RCI) > 1.96 is summarized in Table 3. There was a significant difference between groups (χ²(2) = 7.27, *p* = .026) concerning the number of participants with a reliable reduction in gastrointestinal symptoms with the probiotic and the placebo group not differing significantly (χ²(1) = 0.34, *p* = .562) and with the probiotic (χ²(1) = 4.59, *p* = .032) and the placebo (χ²(1) = 6.77, *p* = .009) group yielding a higher number of participants with a reliable change in gastrointestinal symptoms compared to the no-intervention group. For the gastrointestinal index, there was no significant difference between both groups (χ²(2) = 5.70, *p* = .058). As an exploratory analysis all groups were compared, with the probiotic and the placebo group not differing significantly (χ²(1) = 0.09, *p* = .766) and the probiotic (χ²(1) = 4.93, *p* = .026) and the placebo (χ²(1) = 5.88, *p* = .015) group yielding a higher number of participants with a reliable change in gastrointestinal index compared to the no-intervention group.

**Table 3:** Number of participants with a Reliable Change Index (RCI) > 1.96 as a measure of reliable change in gastrointestinal symptoms and gastrointestinal index per group.

	Placebo (*n* = 22)		No-intervention group (*n* = 23)		Probiotic (*n* = 26)	
	*n*	%	*n*	%	*n*	%
Gastrointestinal symptoms (GSRS)	12	54.5	4	17.4	12	46.2
Gastrointestinal index (GIT)	5	22.7	0	0.0	5	19.2

Note. *n* = number of participants; GSRS = Gastrointestinal Symptom Rating Scale; GIT = Gastrointestinal Questionnaire; GSRS χ²(2) = 7.27, *p* = .026, GIT χ²(2) = 5.70, *p* = .058.

### Effect on psychological stress, physiological stress symptoms, somatization and mental well-being

The 3 × 2 ANOVA yielded no significant interaction of group x time for psychological stress (*F*[2, 68] = 1.50, *p* = .231), somatization (*F*[2, 68] = 2.08, *p* = .132), physiological stress symptoms (*F*[2, 68] = 1.39, *p* = .257) or mental well-being (*F*[2, 68] = 2.05, *p* = .136), as displayed in Table 2.

There was a significant reduction of symptoms for somatization (main effect time: *F*[1, 68] = 39.57, *p* < .001), but no significant reduction of psychological stress (*F*[1, 68] = 0.85, *p* = .360), physiological stress symptoms (*F*[1, 68] = 3.07, *p* = .084) and mental well-being (*F*[1, 68] = 1.98, *p* = .164) over time, as displayed in Table 2.

Moreover, there were no significant group main effects for psychological stress (*F*[2, 68] = 0.84, *p* = .436), somatization (*F*[2, 68] = 2.01, *p* = .142), physiological stress symptoms (*F*[2, 68] = 0.28, *p* = .759), and for mental well-being (*F*[2, 68] = 0.50, *p* = .608), as displayed in Table 2.

### Treatment expectations, perceived improvement

At baseline, there were no significant group differences in treatment expectations (*F*(1,54) = 0.41, *p* = .524) between intervention groups (i.e. consuming fruit bits). Moreover, after treatment, both intervention groups (placebo group *M* = 2.41, *SD* = 0.85; probiotic group *M* = 2.38, *SD* = 0.90) did not differ concerning perceived improvement (*F*(1,44)= 0.01, *p* = .924), and there were no group differences (placebo group *M* = 3.55, *SD* = 0.51; probiotic group *M* = 3.58, *SD* = 0.70) in the perceived general health condition (*F*[2, 68] = 2.67, *p* = .076). Within the placebo group, change in gastrointestinal symptoms (*r* = − .15, *p* = .493), GIT index (*r* = − .13, *p* = .568) and perceived improvement (*r* = − .12, *p* = .607) was not correlated with treatment expectation. Within the probiotic group, perceived improvement was correlated with change in gastrointestinal symptoms (*r* = .47, *p* = .016), while treatment expectation (*r* = − .35, *p* = .080) and GIT index (*r* = − .13, *p* = .522) were not.

## Discussion

In the present first experimental study determining the proportion of the probiotic treatment effect on subclinical gastrointestinal complaints attributable to a placebo effect, we demonstrated a substantial placebo response to fruit bits without probiotic bacteria strains. Individuals suffering from mild to moderate gastrointestinal complaints benefitted from an eight-week intake of a novel probiotic food containing the combination of two bacterial strains (Bifidobacterium animalis subsp. lactis BS01 and Lactobacillus acidophilus LA02, 2.3 × 10^9^ active fluorescent units of per day). However, 46.02% of the reduction of GIT index (*d* = -1.13) was achieved even in the placebo group (*d* = -0.52). There was a comparable reduction of gastrointestinal symptoms due to placebo treatment (*d* = -1.17) and due to probiotic treatment (*d* = -1.08). Explicit treatment expectation was not correlated with change in gastrointestinal symptoms (*GSRS*) and *GIT* index in both groups, but with perceived improvement solely in the probiotic group. We could not demonstrate the efficacy of either probiotic or placebo treatment on perceived stress, somatization, and well-being in participants suffering from subclinical mild to moderate gastrointestinal complaints.

Previous research has already demonstrated placebo effects in the treatment of gastrointestinal complaints in patients suffering from gastrointestinal disorders, with higher response rates in FGID/GBID compared to organic GIT diseases (e.g. 30–40% for IBS and 15–30% for organic GIT diseases)^17^. Similar to the study by Ringel-Kulka et al.^13^ evaluating the probiotic Bifidobacterium infantis 35624, the probiotic fruit bits and the placebo fruit bits were similarly effective in reducing GI symptoms. Moreover, symptom improvement in the probiotic and the placebo groups was greater than in the no-intervention group. Using the RCI as an established measure of clinical significance, participants in the present study were classified as responders (RCI > 1.96) or non-responders. The proportion of responders was higher in the placebo and the probiotic group compared to the no-intervention group. The percentage of participants in the placebo group with a reliable change in symptoms was 54.5% for gastrointestinal symptoms (*GSRS*) and 22.7% for gastrointestinal index (*GIT*). The placebo effect in this study appears to be greater than that reported in the meta-analysis by Enck and Klosterhalfen^17^. This may be because the sample consisted of otherwise healthy individuals with recurrent gastrointestinal complaints, but without manifest gastrointestinal diseases. In contrast, previous placebo research in this area has primarily focused on patients with diagnosed gastrointestinal disorders^17^. Therefore, the gastrointestinal symptoms in the sample were prior to the intervention on average less severe than those of patients with manifest gastrointestinal diseases. Since low severity of symptoms at the beginning of a placebo treatment is a significant predictor of a pronounced placebo effect^43, 44^, this could explain the high placebo response rate on gastrointestinal symptoms in the present study. Furthermore, both groups (probiotic and placebo) received fruit bits with examples of how to consume these fruit bits. Thus, participants were able to just swallow fruit bits solely or use them as add-ons on other potential gut beneficial nutrition as cereals and others. Therefore, both the probiotic and placebo group could have benefitted from regular intake of either 5 g/day mango fruit bits only or from other nutrition supplemented with fruit bits, in contrast to the no-intervention group. Notably, all participants were told to strictly avoid any other probiotic foods during the intervention phase of the study. In addition, participants with subclinical GI symptomatology are less likely to have a strongly altered GUT microflora. Hence, an extra uptake of probiotics might not strongly affect GUT microflora. Rather, both the probiotic and placebo groups might have benefitted from undergoing a daily “intervention” ritual of swallowing fruit bits^45, 46^.

We also explored the role of treatment expectation as the underlying mechanism of the placebo response. Within both groups, explicit treatment expectations were not related to changes in gastrointestinal symptoms and GIT index, indicating that conscious treatment expectation plays a minor role in placebo effects in probiotic treatment. Within the probiotic group treatment expectation correlated with perceived improvement, indicating that treatment expectation is more related to general perception of improvement rather than change in specific gastrointestinal symptoms or the GIT index. However, it could also be an indication of a difference between direct and indirect measurement of symptom improvement.

The lack of a relationship between treatment expectation and changes in gastrointestinal symptoms and GIT index is in line with the limited previous research. In one of the few studies directly assessing treatment expectations in the context of gastrointestinal disorders, Basedow et al.^47^ reported stronger expectations regarding symptom improvement were associated with higher experienced symptom improvement in multimodal therapy of Crohn’s disease. As in our study, the expectation effect was only present when subjectively perceived symptom improvement was assessed but not reflected by pre- to post-symptom change when indirectly determined^47^. However, aside from gastrointestinal symptoms, it is widely accepted that treatment expectations play a significant role in the emergence of the placebo effect^21, 22, 48^. Carlino and Benedetti^49^ demonstrate that a generally positive and rewarding treatment context suggests to the patient that an effective treatment is being conducted, inducing positive treatment expectations, which positively affect the treatment outcome^48, 50^. During the study, we made sufficient efforts to interact with participants empathetically, competently, and kindly to provoke positive feelings and reduce uncertainties or negative preconceptions. Additionally, we took care to create a positive relationship between the research team and participants, as the perception of empathy and competence of the provider also positively influences the treatment outcome^51, 52^. Moreover, information or verbal instructions given by the provider about the effectiveness of the intervention significantly impact the treatment’s success^53^. If patients are informed that the intervention will have a positive effect on their condition, significant symptom improvements can be observed. In this study, a brief text was shown to participants when introducing the test product, which was presented as “fruit crunchies with or without probiotics,” informing participants in a few sentences about the assumed efficacy of probiotics. It was conveyed that probiotics are said to have a positive effect on gastrointestinal complaints due to their direct and indirect effects on gut health. Thus, a positive expectation towards the product was generated. Another factor influencing the placebo effect is the route and salience of medication administration^21^. The placebo in this study was to be taken orally daily, ensuring regular exposure to the product. The open and visible administration of medication positively impacts treatment expectations^48^. In sum, we implemented various strategies to maximize treatment expectations in this study and participants showed a pronounced positive treatment expectation (*GEEE* = 6.96, *SD* = 2.46 on a NRS from 0 to 10) at baseline. However, while implicit treatment expectations, which are directly influenced by the treatment, context may underlie the placebo response, explicit treatment expectations, which are consciously accessible, seem to play a minor role as underlying mechanisms of the placebo response in the probiotic treatment of subclinical gastrointestinal complaints.

We could not demonstrate the efficacy of either probiotic or placebo treatment on perceived stress, somatization, and well-being (secondary outcomes) in participants suffering from subclinical mild to moderate gastrointestinal complaints. This finding stands in conflict with current evidence that placebo treatments can positively affect stress and psychological well-being^54, 55^. This can probably be explained by the fact that during recruitment, as well as in the presentation of the test product, we pointed out the potential positive influence of probiotics on gastrointestinal problems and gut health, but not on perceived stress, somatization and well-being. As a result, we probably missed the opportunity to also generate positive treatment expectations regarding these secondary outcomes. In addition, gastrointestinal tract and food are closely related semantically, which could have activated implicit expectations regarding the change in gastrointestinal complaints but no other outcomes.

Some limitations should be noted. First, our sample of university students is rather selected concerning gender, educational level, and age. While this limits generalizability, we included a subclinical (at-risk) sample and determined reliable change, thus allowing clinical implications. The selection of a neglected, but clinically relevant population with subclinical mild to moderate gastrointestinal complaints without the presence of a manifest and appropriately diagnosed disease might have led to a high fluctuation of symptoms and spontaneous remissions that might have overshadowed the treatment effects of both interventions (i.e., fruit bit conditions). To address this methodological bias, we used a randomized controlled parallel group design including a no- intervention group. By comparing changes in intervention conditions against changes in the no- intervention group, we controlled for confounding effects such as natural symptom fluctuations. Another limitation of this work is that there were no clear criteria by which participants were recruited regarding their gastrointestinal symptoms. It was only specified that the study participants should suffer from “regular gastrointestinal complaints that do not constitute a diagnosed disease”. There is evidence that the severity of symptoms at the beginning of a placebo intervention is a significant predictor of the placebo effect^47^. A low symptom burden at the beginning of the treatment of gastrointestinal complaints is a significant predictor of a pronounced placebo effect^43^. In addition, we cannot fully rule out that the sample might have also been somewhat self-selected with regard to a heightened interest in “natural” remedies for gastrointestinal symptoms. Yet, these individuals are particularly likely not only to suffer from regular gastrointestinal symptoms and being bothered by it, but also to possibly take such probiotics in the expectation of symptom relief. Therefore, future studies should define minimum and maximum cut-off values for participant inclusion. Finally, it should be noted that the actual number of the included participants was much lower than the a priori calculated sample size. While this clearly reduces the power of study, it should be noted that the actual effect sizes for the group x time interaction was greater than assumed a priori (*f* = 0.53). Moreover, the effect size for the difference between the probiotic and the placebo group was very small *(d* < 0.1).

In summary, our findings demonstrate that the treatment effects of probiotic food on mild to moderate subclinical gastrointestinal symptoms and the GIT index are predominantly attributable to placebo effects, possibly explaining why probiotic dietary supplements have become hugely popular for improving gastrointestinal symptoms and gastrointestinal well-being, despite the unclear clinical evidence. Future studies should evaluate the efficacy of probiotic food supplements in clinical populations and possibly using higher concentrations. The role of treatment expectation warrants further investigation as explicit expectations were not found to account for the placebo effects in probiotic treatment.

## Data Availability

The datasets generated and/or analyzed during the current study are available from the corresponding author on reasonable request.
